# Lead Zirconate Titanate Transducers Embedded in Composite Laminates: The Influence of the Integration Method on Ultrasound Transduction

**DOI:** 10.3390/ma16083057

**Published:** 2023-04-12

**Authors:** Nina Kergosien, Ludovic Gavérina, Guillemette Ribay, Florence Saffar, Pierre Beauchêne, Olivier Mesnil, Olivier Bareille

**Affiliations:** 1 DMAS, ONERA, Université Paris-Saclay, F-92322 Châtillon, France; ludovic.gaverina@onera.fr (L.G.); florence.saffar@onera.fr (F.S.); pierre.beauchene@onera.fr (P.B.); 2 Université Paris-Saclay, CEA, List, F-91120 Palaiseau, France; guillemette.ribay@cea.fr (G.R.);; 3 Ecole Centrale Lyon, LTDS, CNRS UMR 5513, F-69134 Écully, France; olivier.bareille@ec-lyon.fr

**Keywords:** structural health monitoring (SHM), embedded lead zirconate titanate (PZT) transducer, carbon fiber-reinforced polymer (CFRP), Lamb waves, laser Doppler vibrometry (LDV)

## Abstract

In the context of an embedded structural health monitoring (SHM) system, two methods of transducer integration into the core of a laminate carbon fiber-reinforced polymer (CFRP) are tested: cut-out and between two plies. This study focuses on the effect of integration methods on Lamb wave generation. For this purpose, plates with an embedded lead zirconate titanate (PZT) transducer are cured in an autoclave. The embedded PZT insulation, integrity, and ability to generate Lamb waves are checked with electromechanical impedance, X-rays, and laser Doppler vibrometry (LDV) measurements. Lamb wave dispersion curves are computed by LDV using two-dimensional fast Fourier transform (Bi-FFT) to study the quasi-antisymmetric mode (qA0) excitability in generation with the embedded PZT in the frequency range of 30 to 200 kHz. The embedded PZT is able to generate Lamb waves, which validate the integration procedure. The first minimum frequency of the embedded PZT shifts to lower frequencies and its amplitude is reduced compared to a surface-mounted PZT.

## 1. Introduction

In the aeronautic domain, aircraft parts need to be inspected in order to prevent accidents. The material fatigue or impacts in composite material parts may induce failures such as matrix, fiber cracks, or delamination [1]. Non-destructive evaluation (NDE) methods [2,3] are already used to inspect accessible aircraft parts but they do not provide information on aircraft integrity in real-time or non-accessible parts. Structural health monitoring (SHM) systems address some of these NDE limits [4,5]. Guided waves are one of the SHM methods able to detect defects, and this study focuses on them.

As presented in several studies, guided wave SHM systems can be used in varying environmental conditions such as temperature [6,7] and moisture [8], or in the presence of vibrations [7] and mechanical loads [9] if the compensation of their effects is applied. In recent studies, PZT-SHM systems are more often bonded to a structure [10,11], but the bonding layer is the principal weakness of a surface-mounted SHM system. Indeed, the thickness and stiffness of the bonding layer affect the coupling properties, and consequently the transduction efficiency [12,13]. Moreover, the bonding layer is very sensitive to environmental conditions (moisture [14] and temperature [7,15]) and deteriorates with aging [16,17]. To overcome such bonding layer issues under environmental conditions and also provide protection from impacts, one solution is the integration of the SHM system directly into the material. Composite materials allow the integration of a monitoring system directly into the material during manufacturing [18,19,20,21,22,23,24,25,26,27,28,29,30]. Moreover, as a co-cured surface-mounted SHM system, the manufacturing process of the composite material can be monitored directly from the beginning of the composite fabrication with an integrated SHM system [30,31]. 

Several studies have already focused on integrating SHM systems into composite materials. The review by Meyer et al. [32] covers various methods of the integration of various types of transducers in laminate composites. The integration is more or less difficult depending on the composites’ manufacturing process and conditions (pressure, temperature) or the fibers’ nature (low or high electric conductivity–carbon/glass) [1]. 

Lots of different transducers can be used in SHM, such as piezoelectric transducers (wafer, a piezoelectric fiber composite (PFC)), electrical resistive or strain gauges, or fiber Bragg grating [1,25]. 

In particular, thin piezoelectric transducers are able to transmit and receive ultrasonic (US) waves. In thin plate-like structures, ultrasounds propagate as guided waves called Lamb waves. Indeed, they offer the advantages of propagating over a long distance compared to bulk waves and are sensitive to defects in the thickness of the structure. While propagating in the structure, US waves interact with damage in the composite. More specifically, Lamb waves can be decomposed into two independent wave motions, namely antisymmetric and symmetric, relative to the mid-plane of the plate [33]. At low frequencies, at least two modes can propagate, namely the first antisymmetric mode A0 and the first symmetric mode S0. The sensitivity of these modes to defects depends on the type of the latter. Guo et al. [34] showed that S0 is sensitive to delamination at the interface where the shear strain is non-zero (pulse-echo testing). In their numerical study, Munian et al. [35] highlight that A0 is more sensitive to delamination located at any interface depth, except in the mid-plane (pitch-catch). In addition, the A0 mode inspection leads to the detection of a smaller defect than the S0 mode because of its shorter wavelength.

The detection of defects by Lamb waves is complicated both by the dispersive nature of Lamb waves and by the coexistence of several guided modes. To facilitate the analysis of the signals and consequently the detection of defects by an SHM system composed of PZT, a solution proposed by Giurgiutiu [36] consists of exploiting the tuning effect of these PZT induced by their geometry. The sensitivity to a given Lamb mode of a bonded PZT in generation and reception depends on the thickness of the composite material, the transducer geometry, and the boundary conditions between the transducer and the composite plate. The frequency-dependent mode damping of ultrasound in the material also influences the excitability, as shown by Mei et al. [37]. Santoni et al. [38] studied the effect of the shear lag of the bonding layer on tuning frequency, introducing the concept of “effective PZT dimension” to account for the discrepancies between the ideal bonding and the actual shear lag load transfer mechanism. 

Two types of piezoelectric materials are usually used: lead zirconate titanate (PZT) and PolyVinyliDene Fluoride (PVDF). Advantageously, PVDF is flexible and its polymer provides electrical insulation. On the contrary, a PZT transducer is more brittle compared to PVDF. However, the embedded PZT is more promising, as shown for instance by Tuloup et al. [24], because an embedded PVDF generates lower signals, which requires a specific measuring system. Moreover, the Curie temperature of PVDF (~130 °C) is lower than the recommended curing temperature of an epoxy thermoset for structural applications (180 °C) [39]. 

Integration studies of PZT in CFRP (carbon fiber-reinforced polymer [40]) laminate focus either on the monitoring of the manufacturing process by the embedded sensor on the resulting mechanical strength of the composite with embedded sensors, or directly on defect detection capabilities of the SHM system. Liu et al. [30] monitored the curing process with embedded fiber Bragg grating (FBG) and PZT sensors and used the embedded system for delamination detection. Andreades et al. [26,27] studied the tensile and fatigue of CFRP material with the electric insulation, They added PZT into their previous studies. Mall et al. [21] studied the difference in monotonic load and fatigue testing of a composite with embedded transducers using two methods, namely cut-out or between two plies. Indeed, they show that the tensile strength, the Young modulus, and the lifetime are not affected by the embedded elements (0.254 mm thick embedded PZT with Kapton). A few recent papers have shown the use of an embedded PZT in laminate CFRP for damage detection. Andreades et al. [18] reported artificial delamination and real impact damage detection with a nonlinear ultrasonic method using an embedded PZT (6 mm diameter and 0.3 mm thick) in a CFRP laminate with GFRP galvanic insulation (0.1 mm thick). Huijer et al. [29] embedded PZT in a CFRP to detect the onset of damages with the method of acoustic emission monitoring. 

Very few papers have compared the transmission and reception capabilities of ultrasonic Lamb waves by an embedded PZT versus a surface-bonded PZT. Feng et al. [23] showed that the amplitude of S0-guided waves transmitted and measured by surface-bonded PZT (DuraAct PZT) is higher than that transmitted and measured by PZT embedded in a CFRP composite, except for one measurement at a given frequency (100 kHz), which is attributed to a tuning mode effect of the surface-bonded PZT. However, the mode tuning itself is not studied in the embedded PZT configuration. Additionally, generation and receiving capabilities are not studied separately in their work. To the authors’ knowledge and at the date of redaction of this paper, no published paper focuses on the tuning effect with an embedded PZT in generation.

This study focuses on the use of an embedded actuator for SHM Lamb waves in a thermoset CFRP composite. Because of its common use in SHM and its high Curie temperature, a PZT disk is chosen for the integration. The aim of this study is to observe the effect of the integration on the PZT transducer excitability. More specifically, the influence of the PZT boundary conditions on Lamb wave generation is studied. The manufacturing and integration methods are first presented. Two methods of integration are tested in order to study different boundary conditions around the embedded PZT. Finally, the tuning capabilities of the embedded PZT in the transmission are presented to determine the effect of the integration on the Lamb wave emission depending on the frequency.

## 2. Materials and Methods

### 2.1. Instrumented Plates

**Figure 1:**
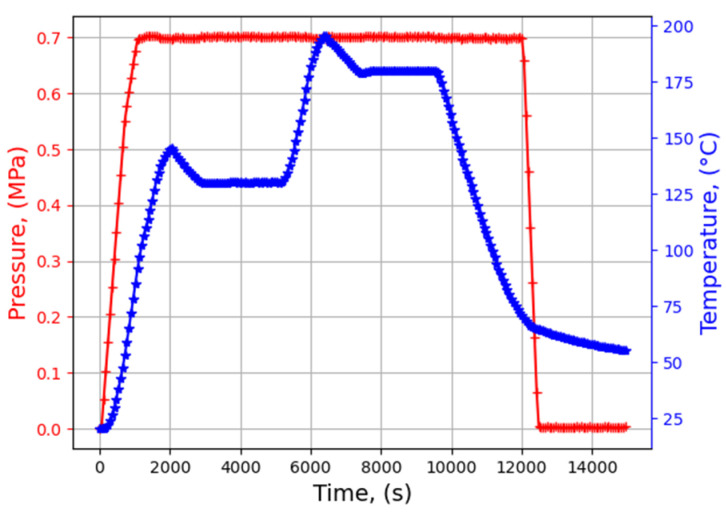
Thermal and pressure measurement during the curing cycle (pressure in red and temperature in blue).

Carbon/epoxy composite plates of T700/M21 are cured in an autoclave. The stacking is [0,0,0,90,90]_s_ (final thickness ~2.63 mm). The curing cycle is drawn from the curing cycle proposed by the supplier of the prepreg. A pressure of 7 bar (0.7 MPa) is applied for 3 h. The cycles of temperature and pressure are presented in Figure 1.

**Figure 2:**
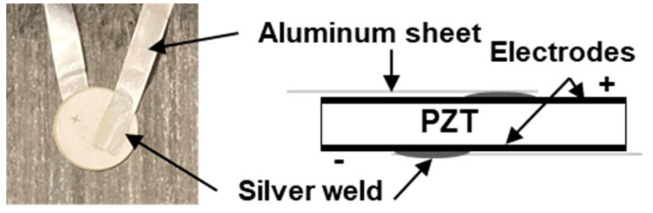
PZT connecting method with a silver weld and an aluminum sheet.

Soft piezoelectric disks in PZT (PIC 255) are selected for integration based on their high coupling coefficient adapted to ultrasonic applications. They are 10 mm in diameter and 0.2 mm thick, which is slightly lower than the prepreg ply thickness of around 0.3 mm. These dimensions were optimized to preserve the PZT integrity during the high-pressure application of the curing cycle. Indeed, the thickness and diameter of the PZT are chosen to minimize the potential bending stress induced by the curing. The disk shape is used to make the source as omnidirectional as possible. The PZT Curie temperature is relatively high (350 °C), thus preventing PZT depolarization and loss of its piezoelectric properties during curing with a relatively high-temperature thermal cycling [41,42]. Attention must be paid to the connection of wires to PZT electrodes. The main drawback of point-like soldering is the resulting punctual load. In this context, a planar connecting method has been studied in a previous work to preserve its integrity during curing [43]. It consists of soldering PZT electrodes to 15 µm thick aluminum sheets with silver paint, as presented in Figure 2. Thin electric insulation is sprayed (silicone spray) on the PZT transducer and aluminum sheets before its integration into the CFRP composite because of the potential electric contact between the embedded transducer and its wires and the carbon fibers of the composite. This insulation prevented power loss.

**Figure 3:**
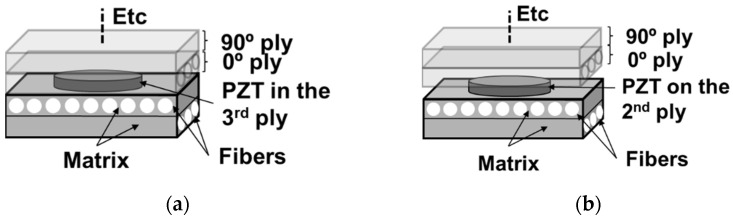
Integration methods tested. (**a**) Transducer directly integrated into the ply and (**b**) transducer between two plies.

Two methods of transducer integration are tested. The first one, named cut-out, consists of cutting out one ply in the shape of the transducer and inserting the transducer directly into the cut-out area in the ply (Figure 3a). The second consists of integrating a transducer between two plies (Figure 3b). In a third configuration, a surface-mounted PZT is bonded with Phenyl salicylate (Salol) to allow comparison with the embedded PZT. Unlike the embedded transducers, the surface-mounted PZT has a wrapped-up electrode.

Five plates are manufactured for this study, one without an embedded PZT, two with a PZT embedded between two plies, and two with a PZT embedded using a cut-out. The integrity of all embedded piezoelectric disks is verified after each embedment by X-ray radiography. The electric insulation is controlled with the measurement of the impedance of all transducers. Only one verification of integrity and electric insulation for each integration method are presented in this article. An overview of the tests presented in this article is given in Table 1. The transducers are positioned in the center of 150x150 mm^2^ plates (75 mm from each edge).

### 2.2. Guided Waves Model

**Figure 4:**
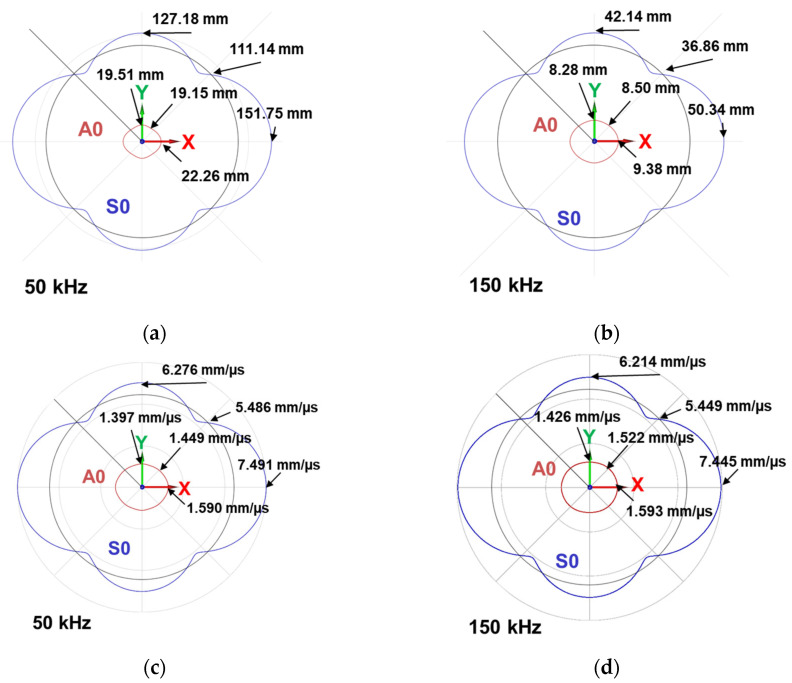
Semi-analytical finite element calculation modes in a polar diagram representation for the experimentally stacked composite, in wavelength (mm) (**a**) at 50 kHz and (**b**) at 150 kHz, and in energy velocity (**c**) at 50 kHz and (**d**) at 150 kHz (2.63 mm of thickness–400 × 400 mm^2^).

A theoretical semi-analytical finite element (SAFE) model available in CIVA^®^ software (Guided waves mode computation—2021 version) [44] is used to compute theoretical wavelengths and mode shapes in the anisotropic laminate composite. Antisymmetric and symmetric modes are called quasi-antisymmetric and quasi-symmetric modes in this paper because the displacement is not purely antisymmetric or symmetric with respect to the mid-plane of the plate due to the anisotropy of the latter. The elasticity coefficients were determined from the comparison between the dispersion curves of the qA0 mode experimentally obtained with a two-dimensional fast Fourier transform (Bi-FFT) and a SAFE parametric study. Wavelength and energy velocity polar diagrams are presented in Figure 4. The X-axis in red matches with the 6 plies at 0° of orientation.

Figure 4 shows that qA0 mode propagation is more isotropic than the quasi-symmetric (qS0) mode despite the anisotropic laminate. Figure 4a,b allow us to estimate the typical size of a detectable defect in this plate depending on the orientation of the plate. Indeed, the minimum detectable defect size is around half of the wavelength; thus, the frequencies need to be chosen accordingly. Figure 4c,d show that there is a 12% deviation at 50 kHz and a 10% deviation at 150 kHz between the higher and lower energy velocity of the qA0 mode. Consequently, the dispersion depends on the propagation orientation of the qA0 mode, and this phenomenon is more visible at low frequencies. Using the qA0 mode thus minimizes the dependence of the propagation with orientation for the detection of a defect.

**Figure 5:**
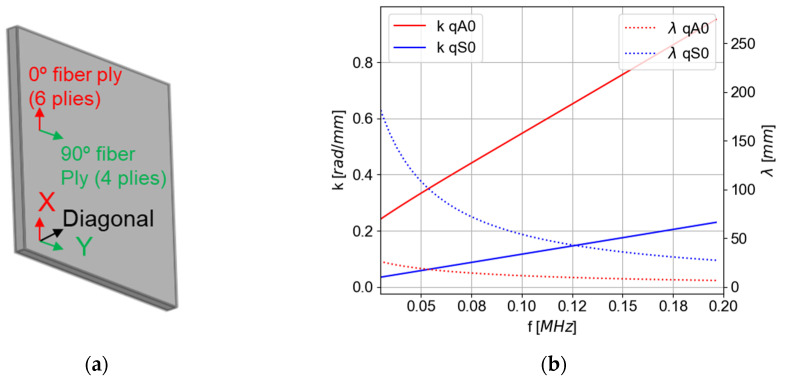
Semi-analytical finite element calculation modes (dispersion curves) in diagonal propagation (identified in (**a**)) in wavenumber (rad/mm) for the composite square plate used experimentally (2.63 mm of thickness–400 × 400 mm^2^) (**b**).

The wavenumber (solid lines) and wavelength (dashed lines) along the diagonal direction of the propagation as a function of frequency, called dispersion curves, are presented in Figure 5. Supplementary Lamb modes appear above 200 kHz in this composite plate. The frequency range is set below 200 kHz to avoid these supplementary Lamb modes and simplify the interpretation of the results in the future use of the embedded PZT for defect detection.

The qA0 wavenumber is 0.334 rad/mm at 50 kHz and 0.762 rad/mm at 150 kHz. The symmetric mode has a lower wavenumber, with 0.087 rad/mm at 50 kHz and 0.178 rad/mm at 150 kHz. The wavenumber estimation allows identifying the Lamb wave modes measured with the vibrometry set-up. In the diagonal mode at 50 kHz, the wavelength is 19.15 mm for qA0 and 111.14 mm for qS0. At 150 kHz, the wavelength is 8.50 mm for qA0 and 36.86 mm for qS0. Consequently, the qA0 mode allows the detection of smaller defects compared to the qS0 mode. For all these reasons, this paper focused on the study of the qA0 mode.

### 2.3. Piezoelectric Integrity Verification

#### 2.3.1. X-Radiography

In a former study, the ability of the X-ray radiography method to reveal cracks in the embedded PZT in CFRP materials has been studied [43]. The set-up is composed of an X-ray source as a microfocus tube (Viscom XT9225DED). The detector is a matrix of photodiodes Perkin Elmer XRD 0821 (1024 × 1024 pixels of 200 µm). The voltage of the acquisition is 100 kV and the current is 50 µA, with 1 s of integration time. The gain of the detector is 1 pF. Ten images are averaged for each acquisition.

**Figure 6:**
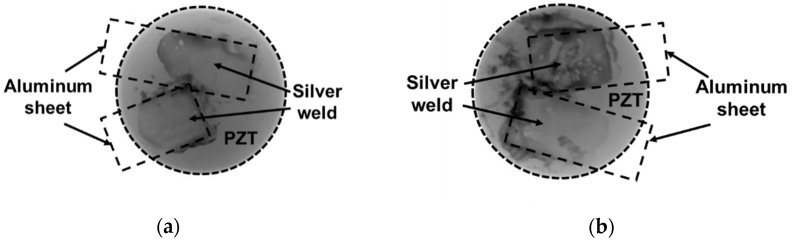
X-Radiography of (**a**) the embedded PZT with the cut-out method and (**b**) the embedded PZT between two plies of prepreg (1 pixel ~20 µm).

Figure 6 presents the pictures of the embedded PZT for both integration methods: cut-out and between two plies. X-ray radiography pictures show the absence of cracks on both PZTs. Visible dark grey zones in the pictures correspond to silver soldering located between PZT electrodes and aluminum sheets.

#### 2.3.2. Electromechanical Impedance Measurement

**Figure 7:**
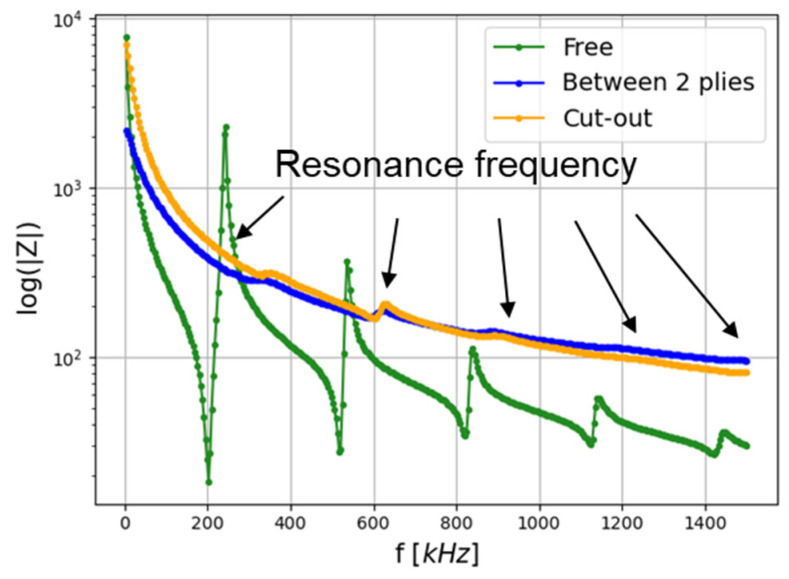
The impedance of the free PZT (green) and the embedded PZT with electrical insulation with the two methods of integration (blue: between two plies and orange: cut-out).

The technique of the electrical–mechanical impedance (EMI) method is widely used in SHM to inspect structures [45,46], but also to monitor transducer properties after coupling to the structure [47], transducer integrity [48], and bonding layer properties [13]. In this article, this technique is used to check the proper isolation of the embedded transducer. The electromechanical impedance is measured with an impedancemeter (4194A Hewlett-Packard), in a frequency range of 100 Hz to 1500 kHz. Measurements are made for one free PZT, one PZT embedded between two plies, and another one embedded in a cut-out. The variability of two free sensors itself is negligible. The impedance measurements are presented in Figure 7.

The free PZT impedance response (in green) displays five resonance frequencies in this frequency range. For both embedded PZTs, the first resonance modes are visible, which proves that the isolation is correct. In fact, if there is a shortcut, the current runs through the composite, which has a lower equivalent resistance than the PZT. Similar acquisitions were made by Feng et al. [23] with integrated DuraAct transducers (Kapton electric isolation). Their electromechanical impedance measurements on their well-insulated transducers showed resonance frequencies with the same shifting to higher frequencies.

Moreover, the first resonance frequency shifts from 200 kHz to 300 kHz were due to the coupling between the embedded PZT and the composite plate. Consequently, no resonance frequency of the embedded transducer is present in the frequency range of interest, namely 30 to 200 kHz. That means the data interpretation is not affected by the PZT resonance frequencies.

#### 2.3.3. Guided Waves Emission

**Figure 8:**
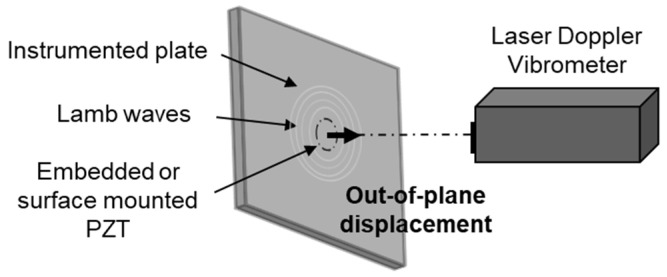
A schematic of a laser Doppler vibrometer bench used to measure the out-of-plane displacement generated by an embedded or surface-mounted PZT.

A laser Doppler vibrometer (LDV) is used as an NDE method to study Lamb wave propagation in plates [49,50]. Therefore, the ability of the embedded transducer to generate Lamb waves is studied with this method. For that purpose, the LDV (interferometer OFV5000 and the sensor head MLV I120) is used to measure the out-of-plane displacement of Lamb waves on the composite surface following the excitation of embedded or surface-mounted transducers (Figure 8). The PZT generates a linear chirp signal from 30 kHz to 200 kHz with a peak-to-peak amplitude of 10 V_pp_.

In order to verify the ability of the embedded PZT to generate Lamb waves, a 100 × 100 mm^2^ area around the PZT is scanned with a step of 1 mm (smaller than the wavelength). A convolution with a burst signal (sinus modulated by a Tukey window with a 0.8 slope) at the frequency of interest is made in order to extract the displacement response at one frequency with a bandwidth [51]. In this article, two frequencies, 50 and 150 kHz, are studied and computed with convolution. At 50 kHz, an 8-cycle sinus windowed by Tukey is employed and the bandwidth at half-height is 10 kHz. In order to have the same bandwidth at 150 kHz, a 30-cycle sinus is used.

**Figure 9:**
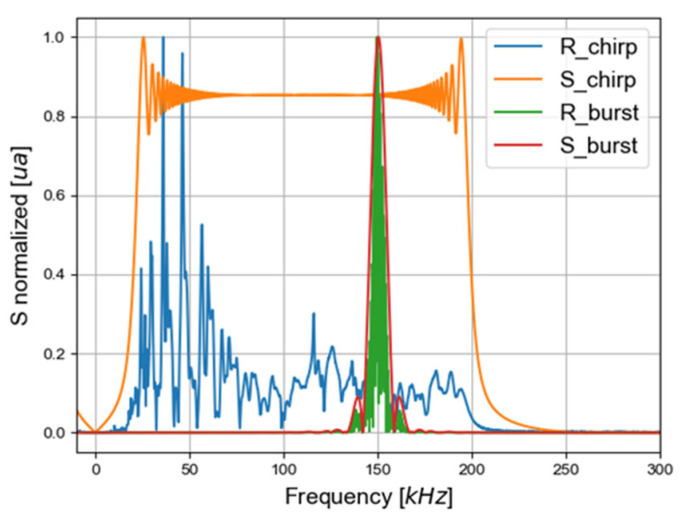
The FFT of the signal registered experimentally with an embedded PZT in cut-out (R_chirp), the FFT of the chirp generated experimentally (S_chirp), the FFT of the signal after convolution (R_burst), and the FFT of the burst used in the convolution (S_burst) at one point in the plate.

The convolution calculation in the frequency domain is:

(1) $$R_{burst}=R_{chirp}.\frac{S_{burst}}{S_{chirp}}$$

where *R_burst_* is the FFT of the registered signal if the emission is a burst, *R_chirp_* is the FFT of the recorded signal after the transmission of a chirp, *S_burst_* is the generated burst signal, and *S_chirp_* is the generated chirp signal. Figure 9 displays an example of the module-normalized FFT, but the post-processing is based on the non-normalized FFT. The R_burst is the FFT of the signal registered if the emission was a burst and depends on the S_burst parameters. After this computation, an inverse FFT is made to convert the signal in the time domain.

**Figure 10:**
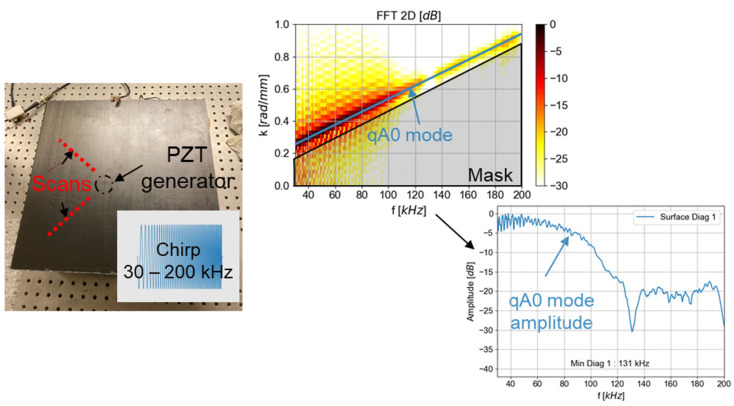
Methodology of qA0 mode excitation extraction (400 × 400 mm^2^ plate).

To quantify the first qA0 mode excitation, a B-Scan of 120 mm is performed in a diagonal direction from 10 mm to the center of the PZT. The B-Scan step is chosen to be smaller (wavelength divided by 6 at 150 kHz) than the minimum wavelength of the excited qA0 mode. The methodology of the analysis is presented in Figure 10. The analysis by Bi-FFT (FFT 2D) [49], which consists in computing a 2D spatial and temporal fast Fourier transform (FFT), is employed to extract the qA0 mode amplitude at each frequency of interest. The mode measured with the vibrometer in this frequency range, which is identified thanks to the previous theoretical calculation (Figure 5). Moreover, the qA0 mode amplitude is greater along the out-of-plane direction, and is thus preferentially measured by the vibrometer. After controlling for the absence of edge effects (reflection) and after applying a mask to suppress qS0, graphics of the extracted qA0 mode maximum of amplitudes are plotted.

## 3. Results

### 3.1. Emission Ability

**Figure 11:**
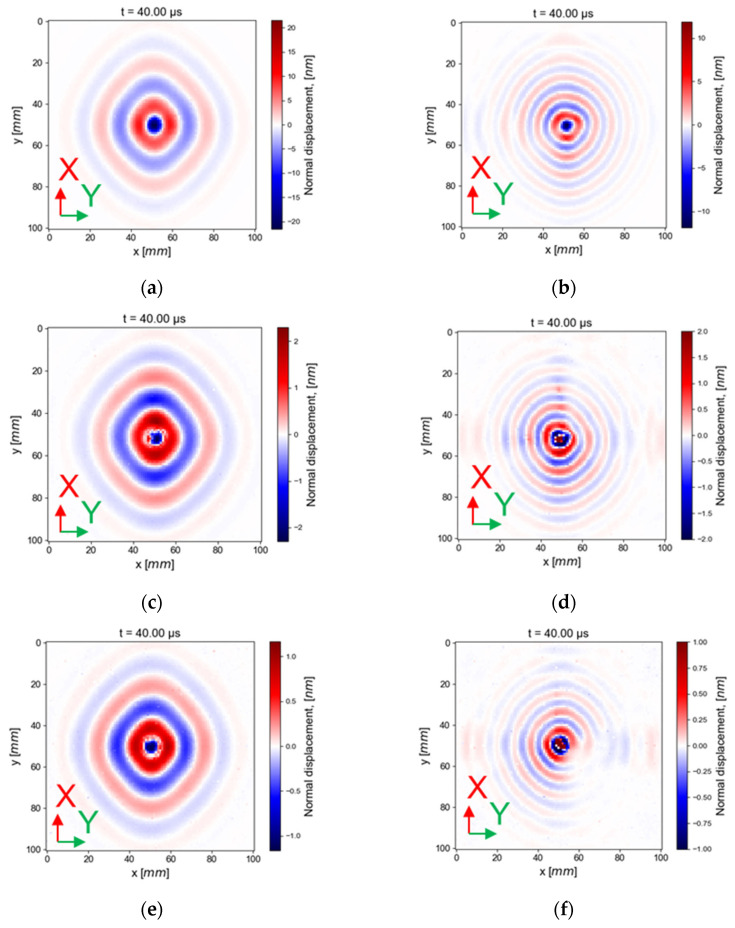
Wave generation, (**a**) Surface-mounted generation at 50 kHz and (**b**) at 150 kHz, (**c**) an embedded PZT in cut-out at 50 kHz and (**d**) at 150 kHz, (**e**) an embedded PZT between two plies at 50 kHz and (**f**) at 150 kHz at 40 µs.

Figure 11 presents the out-of-plane displacement resulting from the generation of a surface-mounted PZT and an embedded PZT. The images are displayed at 40 µs from the beginning of the excitation burst signal to avoid plate edge reflections of the first qA0 mode in the observations (that would be seen at 79 µs, a propagation of 125 mm at 1.6 mm/µs on the X-axis). Two frequencies are displayed. In Figure 11a,c,e the frequency of generation is centered on 50 kHz, and in Figure 11b,d,f on 150 kHz. The X-axis in red matches with the six plies at 0° of orientation.

The images of the out-of-displacement in Figure 11 confirm that the embedded PZT is intact and able to generate Lamb waves at 50 and 150 kHz. In addition, Figure 11 experimentally confirms that the energy velocity of the qA0 mode at 150 kHz barely varies with the propagation orientation contrarily to qA0 at 50 kHz. As expected with the chosen stacking, the wavefront shape of the transmitted wave measured with LDV scanning is in agreement with the theoretical calculation SAFE. Indeed, qA0 energy velocity is greater in the fiber direction (X- and Y-axis).

In Figure 11b,d,f, interferences are visible at 150 kHz. One possible explanation is the superposition of reflected waves with direct waves. The presumed reflected waves appear at around 17 µs (125 mm at 7.5 mm/µs in the X-axis) for the faster mode (qS0 in the X-axis) and at 25 mm from the PZT center, if there is no mode conversion at the plate edges. If there is a mode conversion of the qS0 mode into a qA0 mode on the plate edges, the reflected waves should appear at 41 µs at 25 mm from the PZT center (75 mm at 7.5 mm/µs plus 50 mm at 1.6 mm/µs in the X-axis). The very weak amplitude wave visible in Figure 11b,d,f along the X- and Y-axis could come from reflected qS0 with or without conversion in qA0.

**Figure 12:**
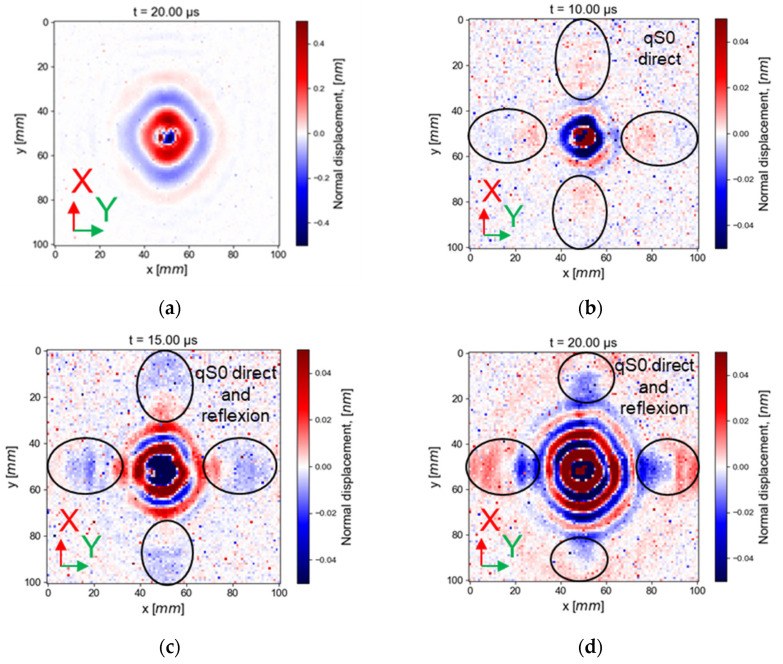
Wave generation with an embedded transducer in the cut-out at (**a**) 50 kHz and at (**b**–**d**) 150 kHz at 10, 15, and 20 µs (first edge reflections for qS0: 17 µs visible at 25 mm from the PZT center).

In order to explain why the reflected waves are more visible at 150 kHz than at 50 kHz, the wavefronts are displayed at an additional time, after the first reflected waves of qS0, in the case of wave generation by an embedded PZT in the cut-out; see Figure 12.

In Figure 12b, the snapshot is displayed at the time corresponding to the qS0 mode arrival to the plate edges. In Figure 12c, the snapshot is displayed at the theoretical time of arrival of the qS0 reflected waves at the abscissa at 10 mm. In Figure 12a,d, snapshots are displayed at 20 µs, which correspond to the later time of the first qS0 mode reflection that could be seen at 25 mm from the PZT center.

At 50 kHz (Figure 12a), the qS0 mode wavelength is bigger than the scanning length; thus, qS0 is not visible. This is consistent with the fact that qS0 normal displacement tends to be zero when the frequency decreases, and is thus not measurable using out-of-plane displacement LDV at very low frequency. At 150 kHz, a larger wavelength coincident with the qS0 mode (a wavelength around 40 mm for the three principal directions of propagation at 150 kHz) is visible in Figure 12b. The qS0 mode at this displayed time is composed of direct and reflected waves. The normal component of the qS0 mode has a higher amplitude at 150 kHz than at 50 kHz. Moreover, the reflected waves are smaller in the diagonal direction, which could be explained by the Maris factor (energy concentrates in the fiber orientation, especially for qS0 mode [52]).

**Figure 13:**
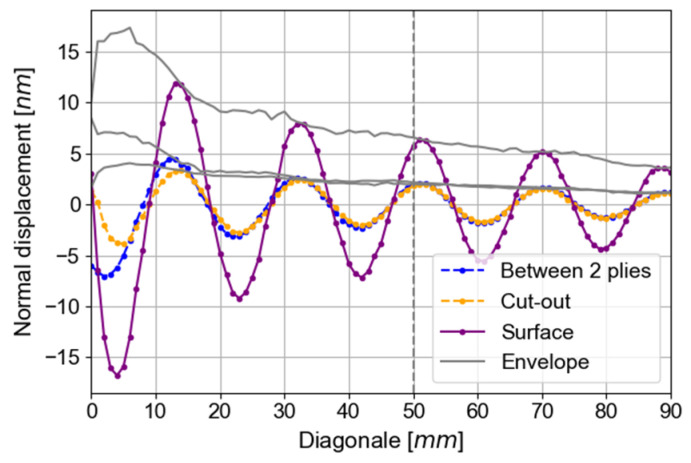
The amplitude of out-of-plane displacements for all tested generations at 50 kHz and 110 µs.

In order to compare the amplitude values with more precision, the out-of-plane amplitudes in the diagonal direction measured for the three methods of excitation are displayed in Figure 13. This direction is chosen to avoid the reflected waves seen in the Y- and X-axis (Figure 12). These measurements are made on bigger plates (400 mm wide and 400 mm long) to avoid wave reflections. In this configuration, qS0 reflection appears at 41 µs (305 mm at 7.5 mm/µs in the X-axis), and qA0 generated by qS0 conversion on the plate edge appears at 110 µs (seen at 25 mm from the PZT center, 165 mm at 7.5 mm/µs and 140 mm at 1.6 mm/µs in the X-axis). Normal displacements are given at a time equal to 110 µs. It is equal to the half duration of the emitted burst (150/2 µs), and the 50 mm traveled (35 µs at 1.5 mm/µs in diagonal for qA0) at 50 kHz.

In Figure 13, the experimentally obtained wavelength is in agreement with the SAFE computation. Amplitudes of qA0 out-of-plane displacements are extracted at least 2λ away from the source for stabilization of qA0 Lamb waves, namely at 50 mm from the PZT center, based on the amplitude of the Hilbert function (envelope). At 50 kHz, with a surface PZT generation, the out-of-plane amplitude is 6.58 nm. The amplitudes measured after the excitation of an embedded PZT in cut-out (2.01 nm) and between two plies (2.16 nm) are equal to the amplitude measured after excitation with a surface-mounted PZT divided by three.

This reduction in amplitude for both embedded PZT generations can stem from the stress applied by the composite on the two faces of the PZT contrarily to the surface-mounted PZT, where stress is applied only on one surface of the PZT due to the bonding.

### 3.2. Quasi-Antisymmetric (qA0) Mode Extraction

**Figure 14:**
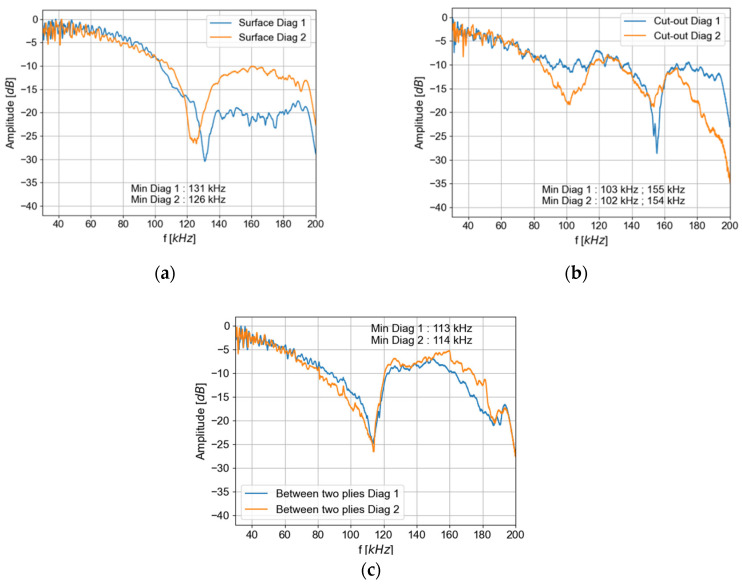
Maximum amplitude of the qA0 mode for a diagonal propagation for (**a**) the surface-mounted generation, (**b**) the generation with an embedded PZT transducer in the cut-out, (**c**) the generation with an embedded PZT between two plies.

The ability to transmit qA0 mode, called excitability, is studied for the three types of transmitters (a surface-mounted PZT, PZT embedded in the cut-out, and PZT embedded between two plies), as the excitability is expected to depend on the PZT boundary conditions. Figure 14 presents the maximum amplitude extracted from the dispersion curve (Bi-FFT) of qA0 mode.

For any given PZT, the minimum excitation frequencies are almost the same for the two diagonals of propagation. On the contrary, these minimums vary with the type of PZT (surface-mounted, embedded in the cut-out, or between two plies).

In Figure 14, the first minimum appears around 130 kHz for the emission with a surface-mounted PZT. The first minimum excitation appears around 100 kHz and a second minimum of excitation appears around 155 kHz for the emission with the PZT embedded in the cut-out. For the PZT embedded between two plies, the first minimum appears at around 115 kHz. Thus, with an embedded generation, the minimum frequency globally shifts to lower frequencies than those observed with a surface-mounted generation. In addition, a second minimum of excitation is detectable in this frequency range for the excitation of a PZT embedded in the cut-out.

For a surface-mounted generation, the amplitude is relatively constant between 30 and 80 kHz (variation of less than −5 dB). For both embedded generations, the amplitude is only constant (+/−2.5 dB) between 30 and 60 kHz.

## 4. Discussion

This article has shown that the Lamb wave amplitude qA0 generated by an embedded PZT is lower than the one generated by a surface-mounted PZT. Moreover, the amplitude of qA0 Lamb waves transmitted by an embedded PZT varies with the excitation frequency depending on the method of integration. It is important to take into account both these phenomena when designing an SHM system for a given application. Indeed, usually, the nature of the composite and the size of the defect to be detected, as well as the distance between transmitter and receiver and their positions (imposed by the geometry of the component), condition the choice of the inspection frequency range. Indeed, if the attenuation of Lamb waves in the composite is too strong, high inspection frequencies may not be compatible with the fixed transmitter–receiver distance (the signal may be buried in the noise). In addition to these usual constraints on the design of the SHM system, the modal excitability also needs to be taken into account. A wrong choice of frequency could lead to too low excitation amplitudes due to the existence of an excitability minimum, as demonstrated in Section 3. In regard to the results, if the protection of the SHM system is essential for the application, and the bonding issues need to be avoided. The choice between integration in the cut-out or between two plies will rely on the desired composite mechanical properties that need to be checked with mechanical tests. Such a mechanical study is outside the scope of this paper.

In addition, in the anisotropic stacking considered in this study, the excitability is expected to be dependent on the orientation of the propagation in the plate. This effect also needs to be considered and will be studied in future works.

## 5. Conclusions

Two methods of integration (between two plies and in the cut-out) have been tested with a new electrode connection method and galvanic insulation. This setup is validated by the verification of the insulation (impedance), the integrity of the embedded PZT (X-radiography), and the ability of the embedded transducers to generate Lamb waves (vibrometry). The integration affects the amplitude of the excited qA0 mode by reducing its amplitude. In fact, the first minimum of the qA0 excitability frequency with the embedded PZT (100 kHz or 113 kHz) appeared below the expected minimum of excitability with a surface-mounted PZT (130 kHz). These observations are closely dependent on the type of integration (surface-mounted, integrated between two plies, or in the cut-out) and more specifically on the boundary conditions between the transducers and the composite plate (bonding layer for a surface-mounted PZT, surrounding composite material for an embedded PZT). Therefore, the authors advise future users of this type of embedded SHM system to characterize their own embedded SHM system with the methods presented in this study, in order to determine the optimal setting of frequency signal of excitation, prior to performing SHM experiments.

This study focused on the out-of-plane displacement of the qA0 mode. It would be interesting to pursue this work with the qS0 mode or focus on the in-plane displacement of both modes. The effect of integration on the PZT excitability needs to be confirmed with other shapes and dimensions of the embedded PZT transducers. Lastly, in future works, the efficiency of this SHM system with PZT embedded with both methods for defect detection and localization will be studied.

## Figures and Tables

**Table 1:** Manufactured plates and carried-out tests.

Plate Dimensions	PZT Location	Carried-Out Tests
150 × 150 mm^2^	Surface	Vibrometry cartography (Section 3.1)
150 × 150 mm^2^	Embedded in cut-out	Vibrometry cartography (Section 3.1)
150 × 150 mm^2^	Embedded between two plies	Vibrometry cartography (Section 3.1)
400 × 400 mm^2^	Embedded in cut-out	Radiography (Section 2.3.1), electromechanical impedance (Section 2.3.2), amplitude comparison (Section 3.1), mode extraction (Section 3.2)
400 × 400 mm^2^	Embedded between two plies and surface	Radiography (Section 2.3.1), electromechanical impedance (Section 2.3.2), amplitude comparison (Section 3.1), mode extraction (Section 3.2)

## Data Availability

Not applicable.
